# Editorial: Resilience of grapevine to climate change: From plant physiology to adaptation strategies

**DOI:** 10.3389/fpls.2022.994267

**Published:** 2022-08-09

**Authors:** Chiara Pastore, Tommaso Frioni, Maria P. Diago

**Affiliations:** ^1^Department of Agricultural and Food Sciences, University of Bologna, Bologna, Italy; ^2^Department of Sustainable Crop Production, Università Cattolica del Sacro Cuore, Piacenza, Italy; ^3^Department of Agricultural and Food Sciences, University of La Rioja, Logroño, Spain; ^4^Department of Viticulture, Institute of Grapevine and Wine Sciences, Logroño, Spain

**Keywords:** abiotic stress, *Vitis vinifera L*., drought, viticulture, sunburn, berry ripening, phenology, breeding

## Introduction

High adaptability of grapevine *(Vitis vinifera L.)* allowed for the expansion of viticulture toward all the main continents over the last centuries, establishing communities whose identity, culture and value system rely on their intimate links with the wine production. However, today the sector is probably facing the most complicated challenges since the post-phylloxera era. Climate change is already posing serious to the industry sustainability, and climate projections seem to predict that worst times have yet to come. In such a scenario, viticulture needs to adapt rapidly to ensure satisfying growers remunerability, keeping intact the links with local traditions and quality of products (Palliotti et al., 2014; Van Leeuwen et al., 2019). In particular, the scientific community is tasked to provide new solutions that can defend this system and solve the main issues of growers under the unpredictability of climatic conditions.

The present Research Topic collect 22 papers, produced by 42 groups spread over 13 different countries. The article collection includes 13 original research articles, eight reviews, and one perspective paper, targeting physiological, molecular and cellular basis of: i. *Vitis vinifera* susceptibility to the most frequently occurring limiting conditions; ii. potential aspects on which agronomic adaptation strategies could rely. Papers included in the collection addressed all the main issues linked to the effects of altered environmental conditions on vine and grape physiology, contributed to fill the specific knowledge gaps, and proposed new solutions and alternatives for the industry.

## Warming trends and vine phenology

One of the main effects of climate change is the compression of grapevine phenology and the advance in harvest dates (Palliotti et al., 2014). Some of the studies included in this collection specifically targeted the description of the effects of rising temperatures on grapevine vegetative and reproductive development rates. First, Pipan et al. proposed different interpretation of weather data in order to calculate heat summation and to model grapevine phenological development. They found that interpolated climate data can be suitable to drive phenological models, but vineyard topography and orography could affect their confidence. In the same framework, Gashu et al. reported that different cultivars exhibit varying sensibility to temperatures, and that the phenological compression observed in specific environmental conditions, can be offset when weather patterns change.

The work by Ausseil et al. highlighted that advancement of ripening due to warming trends is due to an advance in flowering and veraison time, whereas the time-window between veraison and harvest is less affected. In this framework, climatic models can help viticulture to re-arrange cultivars at local or national scale.

However, effects of climate change on phenology do not regard only late season phenology. Increase of spring frost occurrence is for sure one of the unexpected consequences of warming trends. It is linked to the advance in budbreak time recorded in many wine regions due to the increase in temperatures at the end of winter (false springs) and it is one of the most destructive phenomena related to climate change (Poni et al., 2022). In their review, De Rosa et al. tried to make the point about links between cold hardiness, air and soil temperatures, and genetic signaling behind the different varietal behaviors in budbreak time, in order to assist breeders toward the selection of genotypes exhibiting a postponed unlock of bud dormancy, and an increased frost tolerance.

## Understanding effects of climate change on vine and berry responses to identify best counter-actions

Climate change poses new challenges and threats for viticulture, since the composition of berries and quality of wine depend on the main climatic factors, such as water status, radiation, temperature and greenhouse gases (CO_2_) concentration. The effects of climate change are visible on vines and on berries in both primary and secondary metabolism, even altering the relationship between vine phenology and grapevine varietal performance. Temperature increases and more frequent and longer drought periods, are expected shortly in viticultural areas. Even upon the occurrence of small differences in the seasonal mean daily temperature (+ 1.5°C), strong changes on wine grapevine performance and berry primary metabolism may be induced, causing, in warmer environment, earlier onset of phenological events, accelerated vegetative development and sometimes slower (Gashu et al.) or more frequently faster (Allegro et al., 2021) ripening of the berries, which are more intense in red cultivars than in white ones. Even with the increase in atmospheric CO_2_, grape maturity may advance, hastening sugar accumulation and malic acid breakdown, also with differential responses for different clones of the same cultivar (Arrizabalaga-Arriazu et al.). Under higher solar exposure, flavonoids exhibit different sensitivities to degradation, with flavonols being the only compounds that could be positively affected by solar radiation, while anthocyanin depletion is often observed (Torres et al.). In the context of climate change, the night temperatures seem to exert less effects than day temperature on anthocyanin and flavonol accumulation. No effects or very limited ones occur, in fact, on anthocyanins and flavonols in condition of differential night temperatures (Yan et al.). Increasingly frequent drought stress events, have led to evaluate the possibility of alternative water uses, that are often high in salts. In these conditions, the use of rootstocks that are able to mitigate the effect on the scion of high salinity water is necessary as leaf gas exchanges can be reduced and an excess of Cl^−^ and Na^+^ accumulation in the leaves can occur. Anyway, no clear effects on grape berry soluble solids and phenolic compounds accumulation following irrigation with saline water and effects on vine physiology and berry composition should be still elucidated with long-term experiments (Buesa et al.). As temperature and drought increase with climate change, the frequency of extreme thermal events, as heat waves is set to increase. Sunburn is the result in grapevine berries of the complex interplay of environment and grapevine architecture affecting both the local heat impact on the berry surface and the susceptibility of berry. Sunburn damages appear in the berries as a consequence of photooxidative damage that is exacerbated by thermal stress. In these cases, berry response is accomplished through an increased production of antioxidants, HSPs, carotenoids, and polyphenols (Gambetta et al.). With a large variability depending on geographical locations, also the risk of damage due to spring frosts is globally increasing, being a potential risk for grapevine cultivation. Spring frost events, in cold winter regions can cause significant crop losses. To the contrary, warmer regions can be affected by low rates of budburst and lower productivity due to insufficient chilling during winter (De Rosa et al.).

## Identifying resilient plant material to face current and forthcoming vineyard limiting conditions

Genetic diversity of *Vitis* spp. is for sure one of the main points of strength of viticulture and a key-factor for the historical expansion of grapevine cultivation all over the world. The selection of the most adequate plant material for the establishment of a new vineyard is considered the fundament of viticulture long-term adaptation strategies to climate change (Palliotti et al., 2014). Notably, when choosing a specific rootstock, cultivar, or clone, a grower is taking a decision which is going to produce a repeated effect over years from the vineyard plantation to its end-life. Decisions taken today should then consider both current environmental circumstances, as well as those that could take place in the forthcoming 20 or 30 years. Under changing climates and environmental unpredictability, this obviously represents an additional challenge (Palliotti et al., 2014).

Water availability and quality is one of the main issues under climate change conditions. Rootstocks represent the first interface of vine with available water and nutrients. Buesa et al. tested the performance of young Tempranillo vines grafted to the new M1 and M4 rootstock, as compared to the commercial 1,103 Paulsen (1103P), according to a saline irrigation treatment. They found that while showing reduced gas exchanges parameters, M4 was able to preserve better fruit composition than M1 and 1103P.

Grape acidity is one of the fruit composition traits most susceptible to warming trends. Malic acid is indeed quickly oxidized *via* respiration, which is directly dependent to night and day temperatures. Advance of veraison and high temperatures foster the decrease of acidity, which is a key-component of grapes quality for the production of white and sparkling wines (Poni et al., 2018). In their work, Frioni et al. evaluated fruit ripening course of 16 local minor cultivars vs. the locally most common variety. Results highlighted that local germoplasm could hide relevant potentialities in terms of adaptation of viticulture to climate change.

For sure, varietal traits that were considered undesired or of limited interests in the past can be now the focus of renewed interests. In these terms, the work of Sargolzaei et al. assumes high relevance. They went back to *Vitis vinifera* L. domestication sites, in the Caucasus, looking for genotypes exhibiting late harvest or genetic tolerance to pathogens, in order to introduce these accessions in forthcoming breeding programs.

Interestingly, Gashu et al., comparing a set of cultivars in two different sites, reported that varying environmental conditions could stress out or compress differences in the time of onset of veraison, or in malic acid degradation rates.

In terms of plant material, last level for driving vineyard tolerance to environmental pressure is intra-varietal variability. Arrizabalaga-Arriazu et al. tested the effects of elevated air CO_2_ concentration and temperatures on fruit composition of different clones of Tempranillo. They found that, in such conditions, different clones, while exhibiting similar organic acid degradation rates, had significantly different sugars accumulation patterns and anthocyanins accumulation rater.

Overall, a significant number of the works included in this collection focused on genetic resources, varietal selection and rearrangement, and new insights for breeding. Altogether, the authors provided a wide overlook of the relevance of grapevine genetic diversity for the adaptation of viticulture to climate change.

## Modern soil and canopy management in the climate change scenario

In the last few decades, climate change has already been affecting the regional suitability of grapevines. Modeling approaches can be useful to describe the future situation of grapevine cultivation worldwide (Ausseil et al.) being, at the same time, a promising tool to prevent the risks caused by extreme thermal events (Bahr et al.). The availability of accurate climate data is actually necessary in order to evaluate adaptation strategies and to establish how to manage vineyard soil and vine canopy, select vineyard site, choose the most suited cultivar in a particular environment and predict phenological development (Pipan et al.). Quite often, a combination of adaptation strategies provides better solutions, even if only a small number of studies have developed approaches to quantify feasibility and effectiveness of adaptation strategies and have assessed their economic impacts, especially at vineyard scale (Naulleau et al.). Among the modern soil strategies management, in arid regions with unstable climatic patterns, the Direct Root-Zone irrigation (DRZ) could allow economizing water and ensuring grape production through the induction in the vine of the production of deeper roots and the improvement of the photosynthetic rate and the enhancement of grapevine adaptation (Ma et al.). Plant growth-promoting rhizobacteria (PGPRs) are bacterial groups obtained from rhizosphere soil, that can promote plant growth by means of biological control against soil-borne pathogens, biological nitrogen fixation, and root growth promotion (Pii et al., 2015), but also mitigate environmental stresses through different mechanisms. One of the mechanisms involves the production of ACC deaminase, that is able to lower ethylene levels and enhance growth, which is in general reduced by excessive ethylene under environmental stressful conditions, in particular drought. For these reasons, the application of PGPRs could be a promising strategy for mitigate the effects of drought stress. Recent researches demonstrated that the inoculation in the soil of ACC-deaminase producing PGPRs, as a single strain or in mixed combination, can affect vine phytohormones biosynthesis and induce the ROS defense system contributing to the response to the drought stress (Duan et al.). In some grape growing regions, mainly across most of the United States, excessive precipitation events have greatly increased due to climate change, with detrimental impacts on plants and soil in vineyard due to an increase of the erosivity of soils. In these situations, either natural or seeded under-vine vegetation (UVV) can help mitigate many of the problems associated with excessive precipitation, providing vegetative coverage to reduce the force of raindrops, increasing soil organic matter and enhancing soil microbial diversity (Vanden Heuvel and Centinari). Concerning the modern strategies of vine canopy management, in vertically shoot positioned trellis the impact of heat waves and exposure of berries can be mitigated through a partial shading (-60% of solar radiation) of the cluster zone obtained with the application of shading nets, that are able to lower the temperature of 3.9°C in the shaded clusters in comparison to the exposed ones. In combination with water supply, this practice could also avoid berry dehydration during the last part of ripening with beneficial effects on anthocyanins and flavonols, in comparison to fully exposed clusters (Martínez-Lüscher et al.). Interestingly, as reviewed by VanderWeide et al. performing pre-bloom leaf removal to achieve high fruit quality in challenging growing climates seems to be a good strategy, since berry composition significantly improves following pre-bloom defoliation due to the decrease in yield and in bunch rot disease. Ozone (O3) in the troposphere is a highly oxidizing atmospheric pollutant. In addition to climate change, elevated O3 concentration severely affects the growth and development of plants, included grapevine (Blanco-Ward et al., 2021). O3 stress induces the release of large amounts of ethylene by the leaves and canopy treatments with melatonin could significantly inhibit the ethylene response mediated by O3 stress inducing a positive response in photosynthesis and ROS scavenging systems (Liu et al.).

## Emerging methodologies and molecular tools to explore new frontiers in viticulture

One of the key strategies to adapt to climate change (Naulleau et al.) is the breeding and growing of alternative varieties, better adapted to abiotic stresses or with improved aptitudes in acidity, therefore more suitable for winemaking. Toward this end, insight on the diversity of grape solutes known to be influenced by temperature, such as K^+^, Mg^2+^, Ca^2+^, NH^4+^ has been revealed (Bigard et al.) in 12 different *Vitis vinifera* L. cultivars, that were characterized in their berries, as well as their effect in berry acidity. It was shown that a significant genotypic diversity is prevalent in *Vitis vinifera* L. for fruit composition at physiological ripe stage and that parameters determining berry growth and acids accumulation are susceptible of been manipulated by crossbreeding. In this direction, the state of the art of the molecular tools and their usefulness to understand grapevine response to environmental stress, genetics and genomics of grapevine stress tolerance, and how to control and modulate the genome and its expression are reviewed (Gomès et al.). Regarding this very last strategy, the molecular drivers of cold hardiness loss (particularly critical to cope with late frost damage) and the mechanisms that control deacclimation and budbreak to modulate bud phenology are presented (De Rosa et al.), together with their variability in distinct genotypes. On the other side of the equation, heat waves are more recurrent, particularly in warm regions. These are certainly driving heat and water stress and are often associated with berry sunburn, a disorder causing severe yield loss and decline in berry quality. In this volume, a new modeling approach, which integrates functional-structural plant information and management practices over time has been proposed to identify sunburn-reducing strategies in a given vineyard (Bahr et al.). Moreover, the potential and current methods to improve field phenotyping of grapevine to support the characterization of inter- and intra-varietal diversity, as the closing loop stage of breeding, particularly with regards to tolerance to heat and water stress are also discussed (Carvalho et al.).

## Challenges ahead and perspectives

In the short and mid-term, challenges derived from climate change effects (e.g., phenology shifts, decoupling of grape ripening, berry sunburn, heat and water stress, increase of adverse events such as late frost and hail phenomena, occurrence of new pests and diseases, etc.) will remain or even will get aggravated. Nevertheless, the knowledge about adaptation strategies to cope with them is also substantially increasing and will continue to grow, as demonstrated by the collection of works included in this volume, which cover all sort of strategies, from breeding and canopy management to the state of the art of molecular approaches and phenotyping tools. Although not extensively referred to in this volume, the fast development of electronics, artificial intelligence, internet of things, and sensors in general, considered disruptive technologies, may contribute to advanced, unprecedented monitoring of the crop and the final triggering of precision viticulture, aimed at improving the sustainability and profitability of grapegrowing. This may play a significant role in the optimization of resources (e.g., water) as well as in the reduction of chemical inputs for fertilization and spraying.

In summary, while all this research is commendable, the derived knowledge will not become fruitful if effective training and extension activities, to transfer it to the viticulturists and winemakers worldwide are not put in place. This is certainly one of the cornerstones of the effective and successful adaptation of the grape and wine industry to the challenges driven by climate change.

## Author contributions

TF, MD, and CP have made a substantial, direct, and intellectual contribution to the paper and approved it for publication in Frontiers in Plant Science. All authors contributed to the article and approved the submitted version.

## Conflict of interest

The authors declare that the research was conducted in the absence of any commercial or financial relationships that could be construed as a potential conflict of interest.

## Publisher's note

All claims expressed in this article are solely those of the authors and do not necessarily represent those of their affiliated organizations, or those of the publisher, the editors and the reviewers. Any product that may be evaluated in this article, or claim that may be made by its manufacturer, is not guaranteed or endorsed by the publisher.
